# A Blockchain-Based Product Traceability System with Off-Chain EPCIS and IoT Device Authentication

**DOI:** 10.3390/s22228680

**Published:** 2022-11-10

**Authors:** Lulu Li, Huan Qu, Huaizhen Wang, Junyu Wang, Bozhi Wang, Wei Wang, Jinfei Xu, Zhihui Wang

**Affiliations:** 1 College of Computer Science and Technology, Fudan University, Shanghai 201203, China; 2 Zhuhai Fudan Innovation Institute, Zhuhai 518057, China; 3 State Key Laboratory of ASIC and System, Fudan University, Shanghai 201203, China

**Keywords:** product traceability, blockchain, IoT, hyperledger fabric, EPCIS, data secure share

## Abstract

Blockchain-based traceability systems are a promising approach because they are decentralized, transparent, and tamper proof; however, if all traceability data are uploaded to a blockchain platform, it may affect the efficiency or even lead to data explosion. Additionally, it is difficult to guarantee the reliability of the original data source of massive Internet of Things (IoT) devices. Furthermore, when different enterprise nodes adopt different data storage structures, the costs that are associated with data sharing will increase. In this paper, we have proposed a trustworthy product traceability system that is based on hyperledger fabric and Electronic Product Code Information Service (EPCIS), which is not only capable of making products traceable, but it can also authenticate and authorize the IoT devices that are used for data collection. First, we adopted the on-chain and off-chain collaborative management mechanism in order to alleviate data explosion on the chain. Second, we proposed a scheme to authenticate and authorize devices based on blockchain. Third, we complied with EPCIS and Core Business Vocabulary (CBV) standards and provided the EPCIS location discovery service in order to improve the interactivity. Finally, we implemented and tested the proposed traceability system and compared it with the existing research. The proposed solution provides product information traceability, data tamper proofing, data confidentiality, and data source reliability.

## 1. Introduction

Food and drug safety is directly related to human health and safety. With the rapid development of the digital economy, the concept of supply chain visibility for food and drug safety is gaining more attention than ever before. Many countries have formulated laws or regulations regarding the traceability of food and drugs. In 2018, the United States issued the Drug Supply Chain Security Act, which requires enterprises in the whole supply chain to release the traceability information of prescription drugs [1]. In 2015, China issued the Food Safety Law of the People’s Republic of China, which requires food producers and operators to establish a food safety traceability system [2]. In 2019, the Drug Administration Law of the People’s Republic of China proposed that people who are engaged in drug development, production, marketing, use, supervision, and management activities should ensure the authenticity, accuracy, completeness, and traceability of the whole process [3]. Automatic identification technology, such as barcodes, 2D barcodes, radio frequency identification (RFID), and the Internet of Things (IoT) data capture and processing technologies, can record and process various types of information regarding product visibility in the whole supply chain and enable the products to be tracked and traced, which is an effective means to maintain the product quality and safety [4,5]. However, traditional product traceability systems are usually based on centralized data storage architecture, and the information regarding the traceability is usually stored and controlled by a third-party organization. In this type of system, it is difficult to guarantee data transparency and integrity, and the system might present disadvantages, such as single points of failure, ease of tampering with the information, and insufficient credibility [6].

Blockchain is a tamper-proof, distributed, and decentralized peer-to-peer technology that could be used to track and verify digital transactions, and it has many new features, such as distributed data storage, smart contracts, consensus mechanisms, etc. [7,8]. Blockchain technology is one of the most notable innovations of the 21st century [9,10]. It has been applied in various fields, such as the Internet of Things, supply chain management, healthcare, and cross-border transactions [11,12,13]. As it is decentralized, tamper proof, transparent, auditable, etc., blockchain is a promising solution that could be used in order to resolve the problems that are present in traditional traceability systems, and can provide a secure environment for data capture within the supply chain, especially with regard to event data that are created with wired or wireless sensors [14,15].

Nevertheless, the use of blockchain in traceability systems may also face many technical challenges. The following technical problems need to be solved:

First, as the blockchain normally stores a full set of data in each node, if all of the traceability data are uploaded to the blockchain platform, this may lead to an efficiency issue or may even lead to data explosion, since the traceability data in the supply chain can be very large. Furthermore, blockchain data are often open and transparent, which may cause the leakage of business-sensitive information, such as trade relationships, the quantity of goods, etc.

Second, as the supply chain may use a large number of IoT terminal devices to collect and store visibility data, these devices may have security and reliability issues. The simple terminal device cannot run a complex authentication access control strategy, while traditional centralized authorization is subject to risks such as a single point of failures and data leakage. Therefore, it is necessary to establish an intelligent and efficient multi-center IoT terminal authorization and authentication system to guarantee the reliability of data collection.

Finally, if different enterprise nodes adopt different data storage structures and data standards, the costs of data sharing will increase.

In this paper, we have proposed a blockchain-based traceability system with an off-chain Electronic Product Code Information Service (EPCIS) system and IoT device authentication. Our main contributions are as follows:

First, we adopted the on-chain and off-chain collaborative management mechanism, including a hyperledger fabric platform and an on-chain EPCIS repository. The key information that is related to traceability is small in capacity and is not sensitive, and it can be uploaded to the blockchain platform using a smart contract. Large amounts of traceability data can be stored in the mongoDB of the off-chain EPCIS. The blockchain data can be associated with the off-chain EPCIS data repository. All of the participants in the supply chain can securely access the EPCIS through the trusted discovery service and achieve good data interactivity.

Second, we proposed a scheme to authenticate and authorize devices based on blockchain. We have established a unique identity for each device (i.e., device fingerprint). When the client application (APP) is running on the device, the public key and the private key are generated for the APP ID using the asymmetric encryption algorithm, i.e., RSA. We bind and map the APP public key with the unique ID of the current device and record it on the blockchain ledger using the smart contract (i.e., Chaincode). When the client App accesses the EPCIS system, identification verification is executed with the device using the Chaincode. If the device identification verification is successful, then the APP can upload and store the original event data into the off-chain EPCIS repository. This can ensure the data source reliability of the terminal IoT device.

This work focuses on the on-chain storage capacity, data confidentiality, data source reliability, and data interactivity issues, which are important for the product traceability system. The proposed solution adopts an on-chain and off-chain collaborative management mechanism in order to reduce the on-chain storage capacity and to achieve data confidentiality between the enterprise nodes via user registration and the permission management. Additionally, a scheme to authenticate and authorize devices based on blockchain can ensure the data source reliability of the IoT device. Furthermore, the system complies with EPCIS and CBV standards and provides the EPCIS address discovery service, which can improve the interactivity between all of the participants in the supply chain.

The remainder of this paper is structured as follows: Section 2 introduces the relevant technologies and overviews the related research. Section 3 introduces the requirements and the architecture of the product traceability system, as well as its core components and methods. Section 4 describes the experimental validation and the results analysis and discusses future work. Section 5 presents the conclusions.

## 2. Background and Related Work

### 2.1. Hyperledger Fabric

A public blockchain adopts a framework that allows open participation and offers limited throughput, while a private blockchain runs counter to the “decentralization” concept. For enterprise applications, they usually restrict access to a set of authorized participants, which provides higher transaction throughput and low latency and could protect the data privacy that is related to business activities [16,17]. In this trend, many permissioned blockchains have emerged, such as Corda [18], Quorum [19] and hyperledger fabric (also known as Fabric) [20], which are gaining increasing levels of popularity.

Hyperledger fabric was launched by the Linux Foundation in 2015 [21]. At present, Fabric is the most widely used and well-known permissioned blockchain framework. Rauchs et al. recently conducted a survey that showed that 48% of permissioned blockchain projects are built on Fabric, according to the Cambridge Centre for Alternative Finance dataset [22,23].

The advantages of Fabric include its permission control, its modular design, and its pluggable consensus algorithm. It allows entities to conduct confidential transactions through private channels, while the data are only shared among selected participants, which means that it is suitable for enterprise-oriented product traceability systems in which the participants are usually known but not fully trusted by each other.

There are many important components of Fabric that achieve confidentiality, security isolation, and other features. The channel is the key to privacy protection and data isolation, and the smart contract is used to implement detailed business logic.

### 2.2. GS1 EPCIS and CBV Standards

The ISO/IEC 19987 (EPCIS) standard is an important standard of the GS1 architecture [24,25] and it is an international standard that is widely used for product traceability. The EPCIS standard defines the capture interface and the query interface, and it adopts a hierarchical, modular, and scalable design. The EPCIS system is used to store and share all of the visibility data among the enterprise nodes in the supply chain. The application client interacts with the EPCIS through the capture interface and the query control/callback interface in order to collect and access the event data. The ISO/IEC 19988 Core Business Vocabulary (CBV) standard specifies the structure of the vocabularies and the specific values for the vocabulary elements that are to be utilized in conjunction with the GS1 EPCIS standard [26].

### 2.3. Related Work

The product traceability system enables products to be traceable and trackable by recording the various activities of the products in the supply chain. It is an effective means of product quality and safety management. At present, based on the IoT architecture, there are quite a large number of product safety traceability systems [27,28]. The GS1 organization provides a food safety traceability scheme [29] that uses a globally unique traceability code (GS1 code) in order to identify the food products. It provides traceability services for enterprises and provides convenient product traceability inquiries for the regulators and the consumers. The shortcomings of these systems include their centralized storage and their lack of data reliability.

The blockchain-based product traceability system has distributed storage, an inability to tamper with the information, data security, and trustworthiness, which means that it can be used to solve security problems, such as the high cost of sharing supply chain data and the tampering that takes place during transmission, and the system can be used to help consumers to verify the authenticity of the product quality and the safety traceability data.

In 2016, Feng T. et al. proposed a traceability system for the supply chain of agricultural products, which was based on RFID and blockchain technology [30]. This system enables the data collection to be traceable, and the whole supply chain of agricultural products can be transmitted and shared. In 2018, Huang, Y. et al. proposed a scenario-oriented blockchain system called Drugledger, which enables drugs to be traceable and regulated [31]. It is based on UTXO workflow, and it skillfully prunes the blockchain storage according to the expiration date of the drugs. However, a quantitative assessment of the system has not been presented, and problems regarding the system’s flexibility and scalability remain. In 2019, Pamela H. Chua et al. explored the application of hyperledger fabric in the EPCglobal Network with a blockchain platform [32]. This system replaces the EPCIS repository with a blockchain ledger. In 2021, Uddin, M. proposed a blockchain-based Medledger framework to solve the problems that are related to drug traceability [33]. This system stores all of the drug-related activities, events, and transactions. As blockchain data are not easy to delete, the tracing link data will lead to problems such as on-chain data explosion and low system performance, which will be more prominent as time passes. In 2019, Lin Q. et al. designed a food traceability system that was based on Ethereum and the EPCIS [34], and in 2022, Yao Q et al. proposed an agricultural product traceability system that was based on Ethereum and the Inter Planetary File System (IPFS) [35]. Both of these systems use a dual-storage model, and the off-chain storage is used in order to solve the problem of limited on-chain storage space. Nevertheless, the system has open participation, limited throughput, and high latency, and it depends on electronic cryptocurrency. In 2021, Zhang, L. et al. proposed a traceability-related solution for the agricultural product supply chain [36]. Wang L. et al. proposed a framework to track and trace the workflow of the agricultural food supply chains [37]. Zhang, X. et al. proposed a system architecture based on blockchain in the entire grain supply chain [38]. These systems are based on hyperledger fabric and the IPFS. They store the details of the traceability data in the IPFS and store the file IPFS hashes in smart contracts. They also reduce the on-chain storage overhead, but they cannot achieve secure data sharing between the nodes.

As decentralization and smart contracts are not necessary in some application scenarios (e.g., national Grain Cotton Oil supply chain management), centralized databases that integrate with cryptography primitives in order to achieve tamper proofing and auditability also represent a solution [39]. Such centralized databases include Aliyun Ledger DB [40] and AWS QLDB [41]. Recently, hybrid blockchain database systems have been emerging. We can divide the hybrid systems into two types. One type of hybrid system integrates the database features and builds some of the database components on the blockchain, such as FalconDB [42]. These systems usually have limited API and do not support rich queries. As each block stores a transaction record in FalconDB, it is a waste of the blockchain storage resources. The second type of hybrid system adds blockchain features onto the database, such as BigchainDB [43]; however, these systems have limited smart contract functionality and do not support flexible business logic.

In Table 1, note that the traditional traceability systems have problems that are related to data tampering and insufficient credibility. Many scholars have proposed the use of blockchain-based traceability systems. However, a perfect solution that can consider data privacy and security, standardization, system performance, on-chain storage capacity, and so on does not exist. In addition, these systems do not consider the reliability of the IoT devices.

## 3. System Design and Implementation

### 3.1. Requirement Analysis

The system is designed for the following three types of users that are related to the visibility system: enterprises, consumers, and regulatory agencies.

- Enterprise demand analysis

Every enterprise node from the production enterprise to the sales enterprise needs a system to manage and maintain the product information, and the information interaction between the enterprises requires authentication in order to ensure the security of the data information. In order to ensure the ecological balance of the supply chain system, the enterprises also require a traceability system in order to achieve privacy protection. For example, business-sensitive information needs to be encrypted or hidden and cannot be directly exposed.

- Consumer (patient) demand analysis

Consumers are the service objects of the traceability system, and the consumers’ main requirements of the system are that the product information is traceable, credible, and unable to be tampered with.

- Regulatory demand analysis

The responsibility of a regulatory body is to supervise and manage the whole chain process, from the production to the sales of the products, in order to ensure the quality and the source of the products. Regulators need the system to provide information regarding the product traceability that cannot be tampered with. The system simultaneously needs to display more information regarding the products and the related enterprises to the regulatory authorities.

According to the analysis in the previous sections, this system achieves a trustworthy level of product information traceability by combining the hyperledger fabric and the EPCIS systems. The functions that are achieved are as follows:

1. Permission management

The hyperledger-fabric-based permission blockchain requires identity registration and the verification of the enterprise nodes joining the system and imposes certain restrictions in order to avoid the access of malicious nodes. It also allocates different permissions to the different member nodes according to their actual needs, which ensures the security and the confidentiality of the system.

2. Device registration and authentication

A blockchain-based multi-authorization center is established in order to identify and authenticate the terminal devices in order to ensure a reliable source of device identity.

3. IoT data collection

Trustworthy IoT devices record the whole process of production, processing, storage and transportation, sales, and other tracking records. Furthermore, they directly store public data, non-commercial confidential key data and the corresponding EPCIS resource address, and other data through smart contracts and form transaction records in the transaction ledger on the blockchain in order to prevent repudiation. The detailed traceability data regarding the supply chain is uploaded to the EPCIS repository.

4. Support for the enterprises’ independent deployment of the EPCIS system

The enterprises in the production and circulation process produce a large amount of traceable business data. Today, data are an asset, and product data are referred to as “who produces, who owns”. Therefore, the system needs to support enterprises in independently deploying the EPCIS system, which can realize the localized storage of the traceability data that are generated in the product supply chain process. In this paper, we have designed a platform of distributed traceability management and enterprise-level EPCIS deployment architecture, in which enterprises can deploy the EPCIS themselves or by the leading enterprise. Other small and medium enterprises (SMEs) can upload data to the EPCIS of the leading enterprise as participants.

5. Hyperledger fabric and EPCIS collaborative management

We have realized the data interaction between the on-chain database of the hyperledger fabric (LevelDB) and the off-chain database of the EPCIS repository (MongoDB).

6. Information management, query, and verification

The most fundamental function of the traceability system is to provide consumers or regulators with interfaces and applications to query the product information data. The system is required for on-chain and off-chain data classification, data management, the improvement of a friendly exchange for upper-layer applications, and to enable easy-to-use SDKs to interact with the application layer. The traceability system is required in order to ensure that the data are not tampered with and have authenticity and reliability.

### 3.2. System Architecture

**Figure 1:**
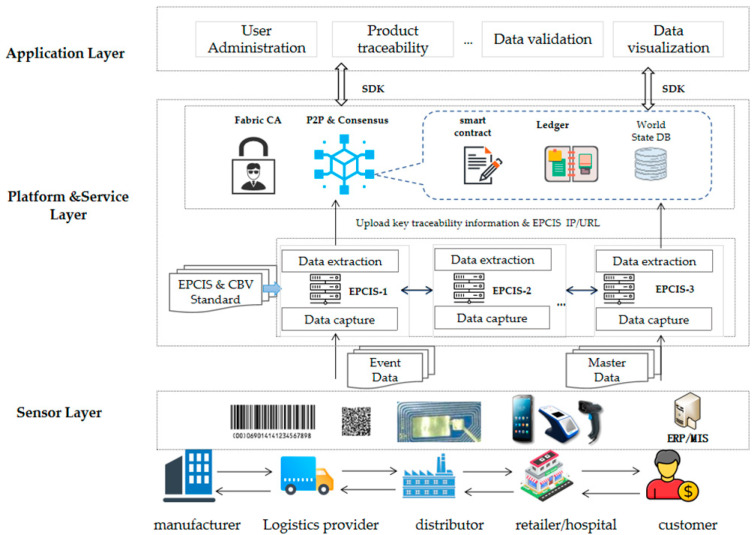
Architecture of the product traceability system based on hyperledger fabric and EPCIS.

In this paper, we have established a trustworthy product traceability system that is based on Fabric and the EPCIS. The system architecture is shown in Figure 1, which is divided into the following layers:

1. Sensor layer

Data collection is usually realized by enterprise nodes that are based on hardware terminal devices (e.g., handhelds) and RFID/barcode technology, and it interacts with the capture interface of the EPCIS. In addition, the applications on the terminal device can interact with the blockchain platform through the SDK in order to achieve device registration, authentication, and to reliably upload the captured data on-chain.

2. Platform service layer

This includes two main parts, namely, the EPCIS and a trustworthy data service that is based on Fabric. The EPCIS module is mainly responsible for capturing the data from the underlying data layer, storing the captured data in the off-chain database of MongoDB, and providing an interface for data query. Given that the data storage format of MongoDB only supports the BSON format, the EPCIS also provides data format conversion and optional subscription services. The EPCIS interacts with the blockchain module through the interaction interface, and it interacts downwards with the perception layer by capturing the API. All of the data structures and the data elements comply with the ISO/IEC 19987 EPCIS standard and the ISO/IEC 19988 CBV standard.

The blockchain hyperledger fabric module is mainly responsible for processing the transaction requests, including the transaction endorsement, the validation, and the consensus services. It interacts with the EPCIS by executing the Chaincode and by storing the data for the key traceability information in the state database, while each validated transaction is stored in the block file system. Similarly, the fingerprint information regarding the device is registered and stored through smart contracts, meaning that Fabric achieves authentication and authorization. In addition, an SDK is provided through Fabric in order to support the various types of application development in the application layer.

**Figure 2:**
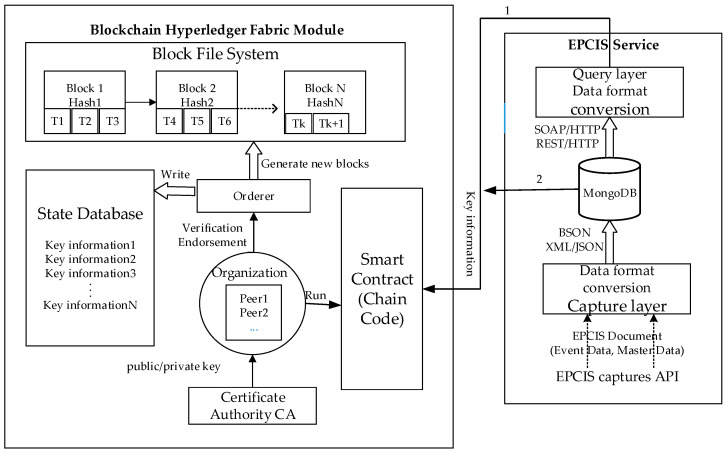
Data interaction between the hyperledger fabric and EPCIS.

The Interaction between the blockchain hyperledger fabric module and the EPCIS module is shown in Figure 2. They interact in two ways. One way is to extract an amount of the key product traceability information data by calling the query interface that is provided by the EPCIS service and uploading it to the blockchain. These data will be stored in the state database as key-value pairs by executing the Chaincode. The second way is to query all of the tracing data of the products from the EPCIS repository (i.e., MongoDB) and to calculate the hash value of the data and upload it to the blockchain. The different enterprise nodes upload the product tracking information, which is packed as a transaction in Fabric. After the transaction, it is added to the block file system.

3. Application layer

The application layer is mainly responsible for user registration and enrollment, it provides a traceability information query service for the consumers and the regulators, and it interacts with the blockchain module through the SDK.

### 3.3. Service Model Based on IoT and Blockchain

The blockchain-based IoT information service model is designed with consideration of enterprise-level distributed deployment.

**Figure 3:**
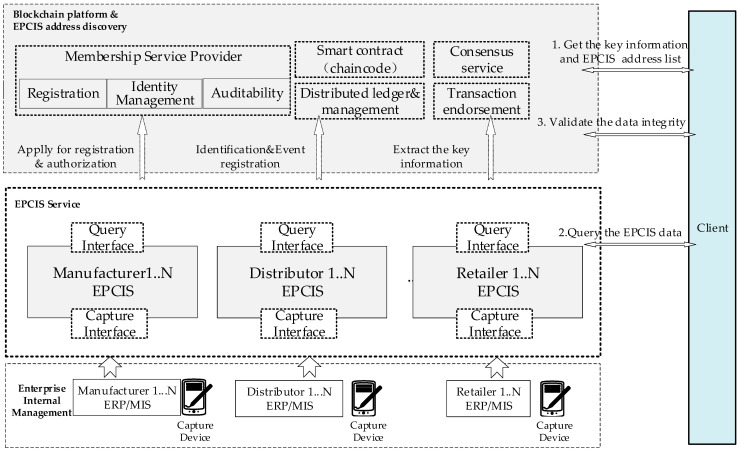
Trustworthy data service model based on the IoT and blockchain.

The service model is shown in Figure 3. It includes an enterprise internal management system, an EPCIS module, and a blockchain-based EPCIS address discovery service. On the one hand, the EPCIS can collect the event data through trustworthy devices with the capture interface. On the other hand, the EPCIS can obtain the types of event data and master data from the enterprise’s internal management system and store them in the EPCIS repository (MongoDB) according to the EPCIS and the CBV standards. Meanwhile, the EPCIS interacts with the Fabric platform and uploads the EPCIS IP or URL to the Fabric platform.

The client can query the product traceability data and can verify the data integrity. At first, the client obtains the key information regarding the product and an address list of the EPCIS according to the EPCIS address discovery service on the blockchain platform. Then, the client retrieves the EPCIS data through the EPCIS query interface. Finally, if the client wants to verify the integrity of the returned data records, they can send a verification request to the blockchain platform again. As the hash summary of all of the data is uploaded to the blockchain as a transaction during the data collection process, the data integrity can be verified.

### 3.4. Implementation

#### 3.4.1. Hyperledger Fabric Network

**Figure 4:**
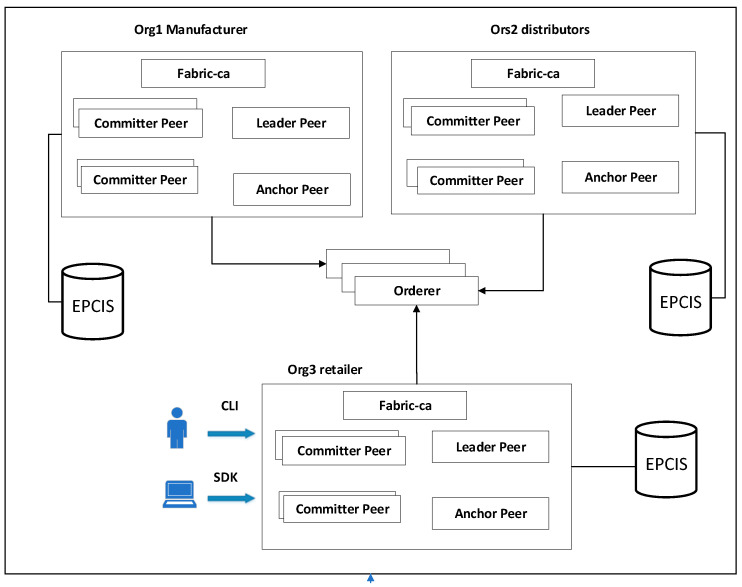
Network deployment scheme.

The hyperledger fabric blockchain network is shown in Figure 4. Three organizations (Org1, Org2, and Org3) have been built in order to represent the production enterprise, the processing enterprise, and the sales enterprise, respectively. Each organization contains two peer nodes, which are responsible for recording and maintaining the ledger and the Chaincode. The Orderer node provides a consensus service, and it sorts and packages the endorsed transactions. Each organization works with a corresponding enterprise-level off-chain EPCIS repository.

- Membership management

The membership management module of the hyperledger fabric is mainly based on the certification authority (CA) mechanism under the public-key infrastructure (PKI) system. When they are applying to join the blockchain, the CA client will apply to the CA server for a unique name and a certificate containing the organization’s name and public key, which complies with the X.509 international standard. The organizations can exchange enrollment certificates (E-Certs) with each other. As the certificate carries information regarding the organizational entities, it is easy to determine whether they belong to the same system and, thus, establish mutual trust. In addition, CA issues a managed transaction certificate (T-Cert), which is used to digitally sign transactions in order to ensure that they cannot be forged. There is also a transport layer security certificate (TLS-Cert), which is mainly used for SSL or TLS communication.

The structure and identity certificates of the three organizations in Fabric are generated through the cryptogenic module by writing the configuration file crypto-config.yaml.

- Data storage and ledger

The transaction is stored in the block file system, and the key-value pair is stored in the state database (i.e., LevelDB). The system sets the primary key as the unique ID of the product, and the corresponding value is a structure in which some key product information is stored (including the production date, the shelf life, the manufacturer, the type of event that occurred, and the EPCIS address).

**Figure 5:**
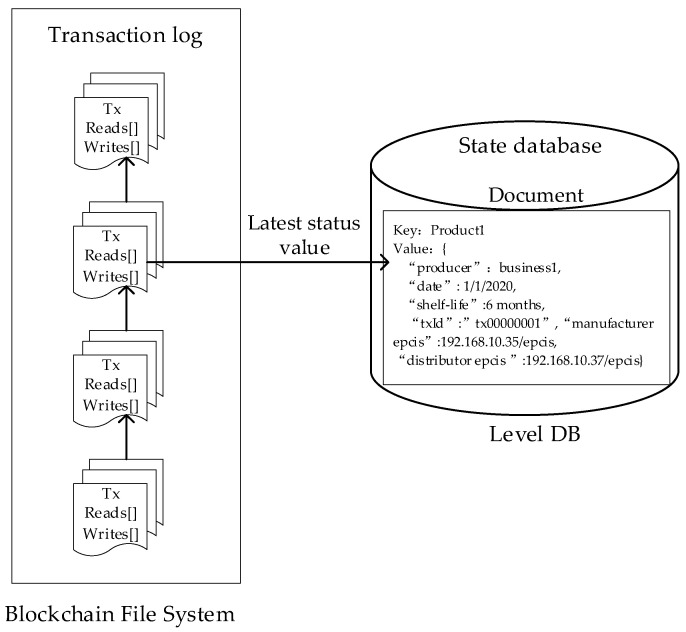
Schematic diagram of the implementation structure of the data storage and ledger.

A schematic diagram of the implementation structure is shown in Figure 5.

- Chaincode

**Figure 6:**
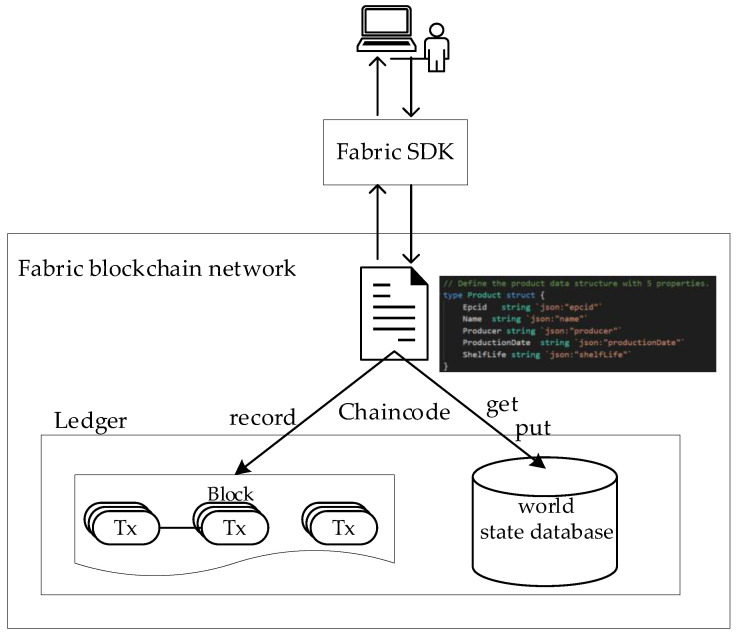
The Fabric network interacts with the client using Chaincode.

A smart contract is the core component of blockchain. We have developed a smart contract in order to realize the business logic of the system. As shown in Figure 6, the Fabric network interacts with the client using Chaincode. The key traceability data of the product is uploaded to the blockchain platform. We have modeled the extracted key traceability data of the product in the Chaincode. The model is a structure that includes the unique product code, the product name, the production date, the event ID, the corresponding EPCIS address information, etc. The traceability data that are generated during the production and the circulation of the product are stored in different EPCIS systems. Therefore, the data field values in the structure are constantly updated and appended. By executing the smart contract, the transaction data are persistently stored in the state database LevelDB. The client can query the EPCIS address list outside of the chain of the product-related enterprises through the product ID. Of course, the structure can be appropriately expanded in order to include more necessary product information according to specific needs.

#### 3.4.2. Blockchain-Based Trustworthy Device Authentication

The EPCIS standard does not specify the way in which the data should be obtained, which can be through the device or from the enterprise’s internal system, such as enterprise resource planning (ERP). When a potential security threat to the IoT device exists, the devices need to be authorized and authenticated first. A multi-center authorization mechanism has been established based on blockchain. The IoT device authentication process is as follows:

1. Device registration

**Figure 7:**
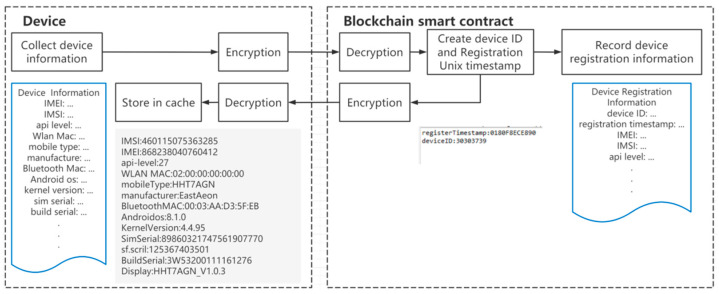
Device registration workflow.

First, the device collects the device information and sends it to the Chaincode. Next, the smart contract gives the device a unique ID and notes the current Unix timestamp. Then, the smart contract records both the device information and the registration information to the blockchain. Finally, the smart contract sends the registration information back to the device, and the device may store it in the cache. The workflow is shown in Figure 7.

2. Real-time tag creation

**Figure 8:**
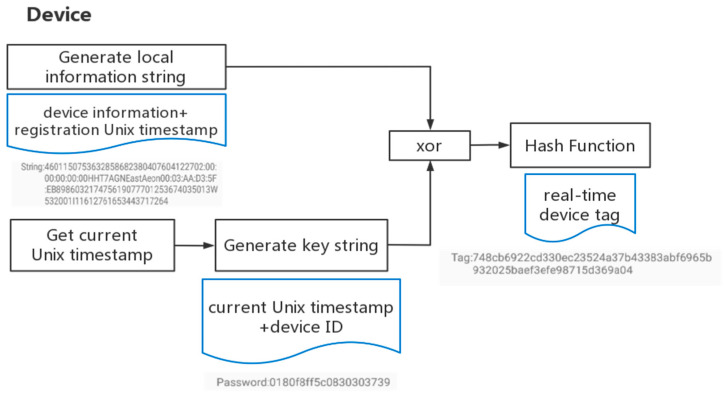
Real-time tag creation workflow.

The device generates a local information string and a key string. The workflow is shown in Figure 8. The local information string includes the device information and the registration Unix timestamp, which is given by the smart contract. The key string includes the current Unix timestamp and the device ID. After an exclusive operation, the device uses a hash function in order to obtain a fixed-length, real-time device tag.

After generating the unique identity, when the client application runs on the device, the public key and the private key are generated for the APP ID through the asymmetric encryption algorithm. In our system, we use the most popular asymmetric encryption algorithm, which is RSA. We bind and map the APP public key with the unique ID (i.e., the fingerprint) of the current device and record it on the blockchain ledger through the smart contract. Then, we can execute device identification verification via the Chaincode.

3. Device identification verification

When the device uploads the data, the smart contract may choose the verification method randomly in order to reduce the load of the system. There are two methods for this, which are as follows:

- Simple verification

As the device ID is included in the transaction header, if the device ID can be found in the blockchain database, the verification is considered to be successful, and the device is valid.

- Check verification

**Figure 9:**
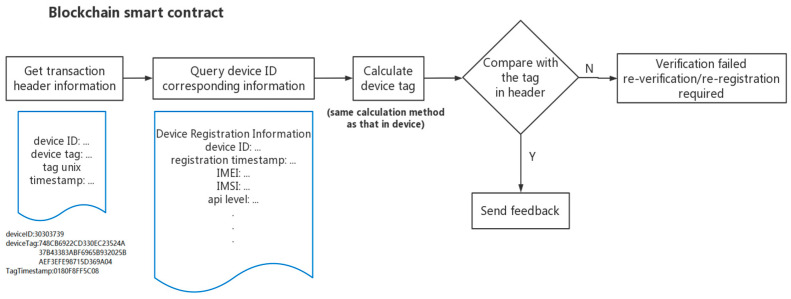
Check verification workflow.

When the device uploads the data, the Chaincode collects the device information in both the header and the blockchain database. The device tag is generated again with the same generation algorithm that was deployed in the smart contract. The device tag that is contained in the uploaded data is compared with the newly generated device tag. If the two tags are consistent, the verification is considered to be successful, and the device is valid. A token will be sent back to the device as proof. The workflow is shown in Figure 9.

#### 3.4.3. Off-Chain EPCIS

In order to ensure interactivity, we have adopted the ISO/IEC 19987(EPCIS) and the ISO/IEC 19988 (CBV) standards, which define the standardized data elements, structures, and formats.

The EPCIS system in our work is based on the EPCIS v1.2 under the open-source project Oliot of KAIST University [44]. According to the EPCIS standard, the master data are composed of the vocabulary, the elements, and the master data attributes. The data structure that we have designed is shown in Table 2.

The EPCIS repository (MongoDB) is mainly responsible for storing four types of event information (EPCIS event). The specific meaning of each of these is shown in Table 3.

The manufacturers, the distributors, and the retailers can organize the product basic data, the enterprise basic data, and the event data into the structure of the EPCIS standard, according to the standard vocabulary that is defined by CBV, and upload it to the EPCIS through the data capture interface (Capture). The users can use the query interface method that is defined by the EPCIS standard to query the EPCIS repository (corresponding to MongoDB) in order to obtain detailed business information. The EPCIS event data structure is shown in Table 4.

#### 3.4.4. Data Interaction between Hyperledger Fabric and EPCIS

The interaction between the hyperledger fabric blockchain module and the EPCIS service module includes the following three operations:

1. Upload the product key traceability information to the state database.

By invoking the query interface that is provided by the EPCIS, we extract some key traceability information and upload it to the blockchain. After the Chaincode is executed, it is stored in the state database (LevelDB). The pseudo code of the algorithm is shown in Algorithm 1.
**Algorithm 1.** Process of uploading the key traceability information to the state database.**Description****Uploading Product Key Traceability Information to the State Database**Input: Output: 1. 2. 3. 4. 5. 6. 7. 8.Extract the key traceability  struct Product { }; Product = json.Marshal (input); //Convert data format stub.GetState (Product); if (err != nil) return; else stub.PutState (Product); //Write information to the status database end if

2. Upload the hash values of the product detail traceability data

We upload the hash values of all of the product traceability data from the EPCIS repository (MongoDB) to the Fabric, instead of the original traceability data. The hash abstract of these data will be recorded in the block as a transaction. Because the hash algorithm has the characteristics of anti-collision and one-way irreversibility, it can ensure the reliability and the integrity of the product data. The pseudo code of the algorithm is shown in Algorithm 2.
**Algorithm 2.** Uploading the hash value of the product detail traceability data.**Description****Uploading the Hash Value of Product Detail Traceability Data**Input: Output: 1. 2. 3. 4. 5. 6. 7. 8.Product information stored in mongodb database Hash (product data); //the hash value of the product detail traceability data if (transaction != nil) endorsing; //The endorsement node endorses transactions consensus service; //Ordering nodes order the transactions if (err == nil) committing; //validate and commit the transactions end if; write into block;

As the state database LevelDB stores the data in the form of key-value pairs, the EPC (such as SGTIN) of the product can be used as the unique key, and the other master data and the event data that are related to the product can be used as the values. As the extracted key traceability information on the chain is open and transparent to the enterprise nodes in the same channel, we do not upload or disclose the business-sensitive information of the enterprise, in consideration of data confidentiality.

#### 3.4.5. Collection of Reliable Data Source by IoT Device

There are two ways in which the client application of the device can collect traceability data. One is to directly upload the collected event data to the blockchain via an IoT device. However, as has been mentioned earlier, this will lead to issues with the on-chain data storage capacity. The second way is to call the capture interface of the EPCIS through the APP application of the device, upload, and store the original event data in the off-chain EPCIS.

**Figure 10:**
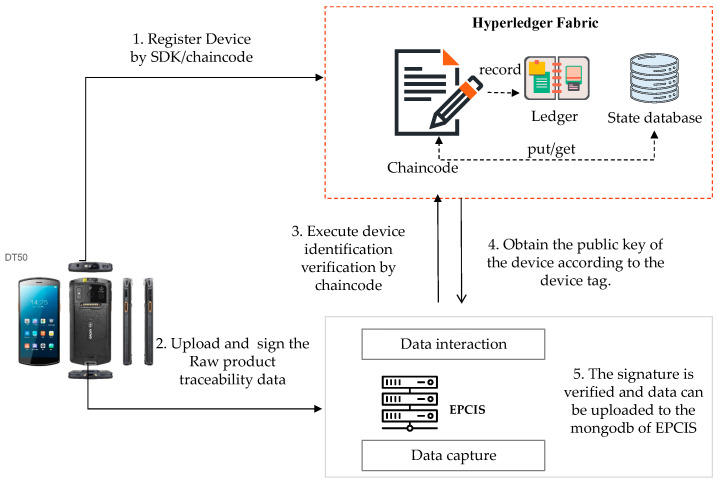
The process of collecting reliable data sources with an IoT device.

In this work, we have used the second method. The process of collecting reliable data via a trustworthy IoT device is shown in Figure 10.

Step 1: Register the devices on the blockchain. This step has been described in detail in Section 3.4.2.

Step 2: The client App accesses the EPCIS system and submits a request. The request data include the unique identity of the device (the device tag), the collected traceability event data, and a signature with a private key.

Step 3: The EPCIS system interacts with the blockchain via the blockchain SDKs and the Chaincode. The EPCIS can obtain the public key of the client APP that is running on the device by calling the stub, the GetState (the device tag) of the Chaincode.

The Chaincode executes the device identification verification.

Step 4: The blockchain platform returns the App public key address according to the unique device tag (i.e., the fingerprint), and the EPCIS system performs the signature verification through the APP public key.

Step 5: If the signature is verified, this means that the current device has been registered in the blockchain and its identity is trusted. At the same time, as the data that are submitted with a trusted device are signed with the private key, it can be confirmed that the data have not been tampered with. After the IoT device is proved to be reliable, it may upload the event data to the EPCIS. Then, the product traceability event data are stored in Mongodb of the EPCIS.

#### 3.4.6. Client-Side Process of Product Traceability Query

**Figure 11:**
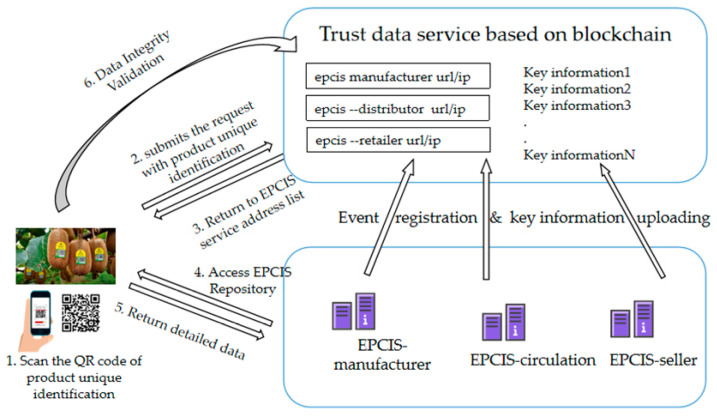
Product traceability resolution process.

The process of a client querying the product traceability is shown in Figure 11.

The steps are as follows:

Step 1: The client scans the QR code of the product’s unique identification.

Step 2: The client submits the GS1 code identification to the blockchain platform.

Step 3: The blockchain platform parses and discovers the EPCIS information service list address corresponding to the code by querying the state database (StateDB) through each link and returns the key traceability data.

Step 4: The consumers access the EPCIS in order to obtain detailed event data regarding each link.

Step 5: The EPCIS system returns the detailed traceability data.

Step 6: If the consumers doubt the returned information, they can submit the obtained event information to the blockchain platform and check the corresponding transaction through the hash value in the chain transaction ledger in order to ensure that the data are credible and have not been tampered with. The specific process is shown in Figure 11.

Incidentally, when the users query the detailed off-chain traceability data according to the address of the EPCIS, the EPCIS systems, which are deployed independently by the enterprises, can set access permissions according to the application scenarios. However, this is not within the scope of this study.

## 4. Results and Discussion

### 4.1. Experimental Environment and Deployment

In order to ensure interactivity, we have adopted the ISO/IEC 19987(EPCIS) and the ISO/IEC 19988 (CBV) standards, which define the standardized data elements, structures, and formats.

The off-chain EPCIS in this system is based on the deployment of EPCIS v1.2 under the open-source Oliot project of KAIST. The equipment and the environment configurations that have been used in this experiment are shown in Table 5, Table 6 and Table 7.

By interacting with the MongoDB database through the EPCIS query interface, one can query the detailed product traceability data of the chain. The key traceability information of the products on the chain is queried by executing the Fabric’s smart contract (Chaincode).

The block header stores the hash values of the previous block and the current block, and the block body stores the transaction information, i.e., the hash digest of the product traceability data. According to the digest, the integrity of the traceability data can be verified.

**Figure 12:**
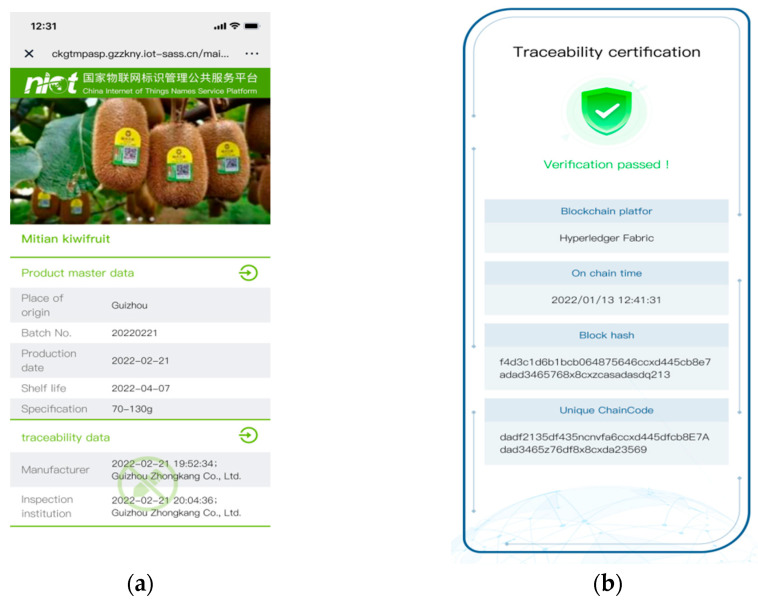
The traceability information shown on the mobile phone. (**a**) Product traceability information; (**b**) Data integrity validation.

We applied our research results in Guizhou City Agricultural Technology Co., Ltd. of China, and the data were guaranteed to be tamper proof and asynchronously verifiable, based on blockchain, as shown in Figure 12.

### 4.2. Performance Analysis

We have adopted the Hyperledger Caliper [45] in order to conduct performance testing, which included the determination of the transaction success rate, the transaction throughput TPS, the response time, etc.

The systems displayed no transaction failures when the request rate was from 100 times/s to 1000 times/s. The reason for this is that we used the different EPCIS as the key in the state database, and the transactions had no conflicts between them. However, according to the literature, if a client payload accesses the same key value in the state database, it may have a high transaction failure rate in the Fabric validation phase [46,47,48].

The system throughput was about 100 tps when the request rate was 100 times/s. As the rate of request increased to 1000 times/s, the throughput could reach 1000 tps. After that, the server was saturated, and the throughput converged to 1000 tps.

**Figure 13:**
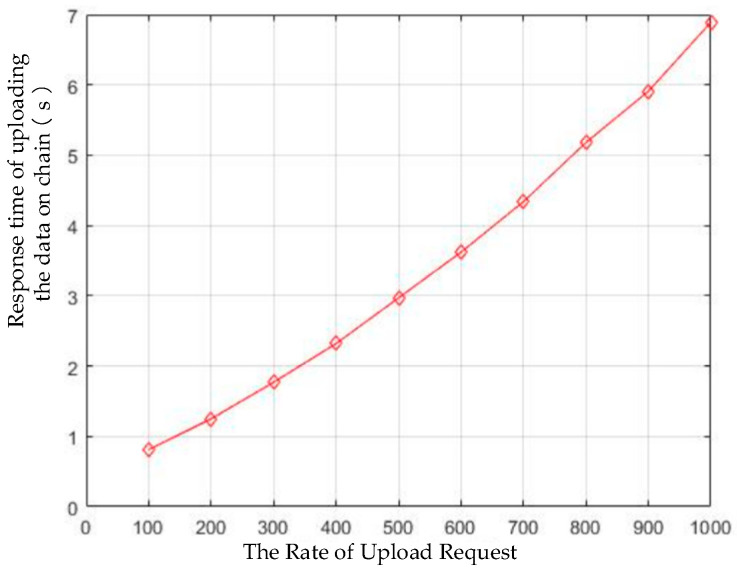
Response time of product data uploaded to the blockchain.

We tested the product information upload response time, and the results are shown in Figure 13. We can see that, when the number of upload requests increased from 100 times/s to 1000 times/s, the response time increased from approximately 0.8 s to 7 s. This is because the consensus process took most of the time (that is, the Fabric ordering service). If the number of transaction requests continues to increase, the transactions in the network will be regularly packaged into multiple blocks. The block needs to wait in the queue and complete the transaction through the Fabric’s execution-order verification (e-o-v) process, thus, increasing the latency. When the number of requests is greater than 1000 times/s, the delay will exceed 7 s; however, we believe that this concurrency is enough in the blockchain scenario.

**Figure 14:**
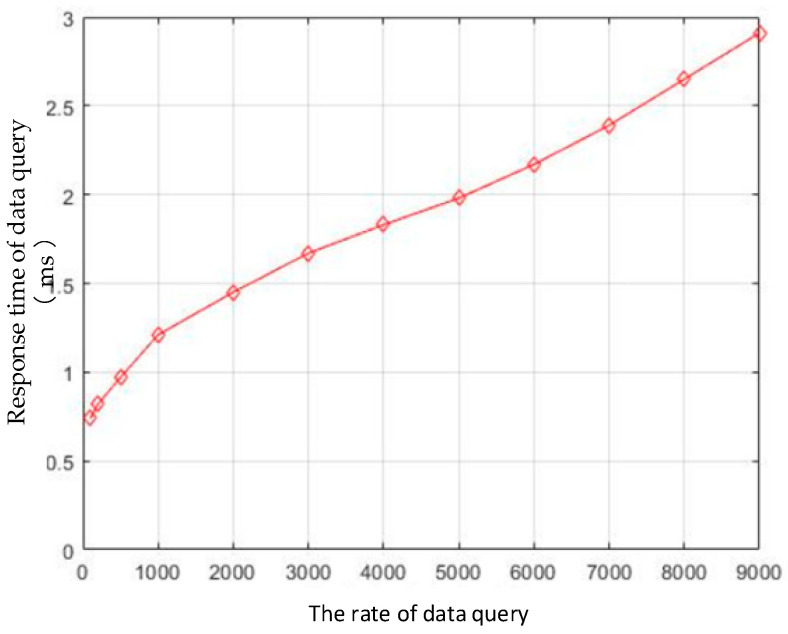
Response time of product data query.

We tested the response time of the data query by increasing the number of calls to the Chaincode by the application. The test results are shown in Figure 14. When the query request rate increased from 1000 to 9000 per second, the response time of the information query slightly increased from 1.2 ms to 2.8 ms, which was mainly due to the network transmission overheads that were caused by calling the Chaincode. This is because the query operation of the state database in the Fabric blockchain network will not generate a piece of transaction information to be recorded in the block, meaning that it does not involve consensus and block processing, and the overall response time and the information uplink response are faster.

### 4.3. System Comparison

We compared the proposed system to other related work, including blockchain-based traceability systems, centralized databases that were integrated with cryptography primitives, and hybrid blockchain database systems (Table 8).

The systems [30,32] store all of the traceability data on the blockchain, which may lead to problems that are related to on-chain data explosion and data confidentiality. The studies [34,35] adopted the dual storage model of “Blockchain + off-chain” and proposed traceability solutions that were based on the Ethereum platform and IPFS. System [35] is open participation and transparent, due to the lack of permission management. System [34] designed a data access control policy between the enterprise nodes based on smart contract; however, the system has limited throughput and long latency (the upload time was tens of seconds). Additionally, these systems depend on electronic cryptocurrency, and, if gas is consumed, the contract will fail to be executed. Our work is based on hyperledger fabric and the EPCIS system. Fabric is more adaptable to the product traceability systems as it has some beneficial features, such as the efficient consensus mechanism, membership management, and rich-interactivity SDKs. The literature of [36,37] are also based on hyperledger fabric. The detailed traceability data is stored in the IPFS, and the hash value of the data is stored on the chain. These systems also reduce the on-chain storage. However, as only the hash values are stored on the blockchain, the public non-sensitive data are difficult to share, and the non-adjacent enterprise nodes that are in the supply chain cannot achieve secure data sharing conveniently.

The literature of [40] makes use of cryptography primitive technology. Therefore, it can ensure data tamper proofing and auditability, and it can have good performance with high throughput and low latency. However, a centralized database should not be used to manage the data flow of upstream and downstream enterprises, especially for cross-border supply chain management, as it is usually not managed by one organization. The literature of [43] involves hybrid blockchain database systems with high performance, as it starts from the distributed database and adds blockchain characteristics. The permission system enables configurations ranging from private enterprise blockchain databases to open public blockchain databases. However, it has a limited smart contract function and does not support flexible business logic. It also does not consider the data reliability of an IoT device.

The proposed solution provides the traceability of the product information. It has advanced features, such as less on-chain storage, data tamper proofing and data source reliability, data confidentiality, and data interactivity. It also can achieve high throughput and low latency.

Less on-chain storage: As the data that we upload to the chain are the key product data, these data are structured text data, usually occupying a small storage space. Detailed traceability data, including product pictures, are stored in the off-chain EPCIS. Therefore, our scheme reduces the data overhead on the chain and alleviates the problem of data explosion on the chain while realizing the data interaction between nodes.

Data tamper proofing and data source reliability: As the blockchain data are difficult to tamper with, it eliminates the impact of the stakeholders on the authenticity of the information and prevents manual tampering. At the same time, we also ensure the reliability of the data source by authenticating the identity of the IoT devices. Only the APP that is running on the authenticated IoT device can upload and store the original event data into the off-chain EPCIS repository.

Data confidentiality: Based on hyperledger fabric, our system registers and verifies the identity of the enterprise nodes that join the system. It has the permission management function, which can prevent malicious nodes from accessing it. All of the participants can only access the transactions that they have access rights to. By designing smart contracts, we upload the key product data and the access address of the EPCIS, which does not involve any sensitive information.

Data interactivity: The system complies with EPCIS and CBV data interaction standards. Using smart contracts, the key data and off-chain EPCIS address are uploaded to the blockchain. The EPCIS address discovery services are implemented based on the blockchain in order to provide secure access between the non-adjacent nodes. It has good, secure data interactivity.

### 4.4. Limitations and Future Research Directions

7. Hyperledger Fabric Transaction Mechanism Study.

Fabric’s transaction process uses execution-ordering-validation (EOV) in order to achieve concurrent transactions and to improve throughput. As far as we know, it is the only permissioned blockchain that currently achieves concurrent transactions. However, it has a high transaction failure rate in the case of contention for key-value accesses in a state database, which has been analyzed in detail in the literature [46,47,48]. It is worth studying how the failure rate of the Fabric transactions might be reduced in the case of high concurrency, such as the establishment of a caching mechanism for reading and writing the collections that are simulated by the transactions, and concurrency control by multiple versions in the sorting process.

8. On-chain and off-chain semantic link.

To date, no product traceability system exists to establish a semantic link between the on-chain data and the off-chain data. In the literature [49], a hybrid database has been studied in relation to how the key semantic data might be stored in block transactions and how a semantic link between the on-chain data and the off-chain business data might be achieved without the redundant storage of the same data in the on- and off-chain. Then, the users can obtain the on-chain and the off-chain data by requesting once and simultaneously verifying the data integrity. This can further reduce the data storage capacity and can improve the query performance. Thus, the study of on-chain and off-chain semantic links is very important and meaningful.

9. Optimize query of on-chain data.

Combinations of AI and blockchain are emerging. AI-based learning indexes have been applied in databases in order to optimize the data query [50]. In the future, blockchain can be combined with AI-based adaptive index technology in order to make the system more comprehensive and efficient. For instance, it can enable aggregation queries, statistical queries, and other functions. In addition, considering the characteristics of the IoT object identifiers and the event data, by building an efficient index for on-chain data, the speed of the data transaction queries could be improved.

## 5. Conclusions

In this paper, we have proposed a trustworthy product traceability system based on hyperledger fabric and EPCIS, which is not only capable of making products traceable, but it can also authenticate and authorize the IoT devices that are for data collection. The proposed system provides product information traceability, data tamper proofing, data confidentiality and reliability.

In the proposed system, the non-sensitive key information that is related to traceability is uploaded to the blockchain platform using Chaincode. Large amounts of traceability data are stored in the off-chain EPCIS repository (mongoDB). By providing a trustworthy discovery service to locate the EPCIS resource for clients, the blockchain data can be associated with the off-chain EPCIS data repository. The identity of the IoT devices in the traceability system are authenticated and authorized in order to ensure the reliability of the data source. The APP that is running on the authenticated device can upload and store the original event data into the off-chain EPCIS repository. All of the data elements and structures comply with the GS1 EPCIS and CBV standards in order to enhance the data interactivity between the participants in the supply chain. Taking kiwi fruit traceability as an example, we have established an application demonstration in an enterprise in Guizhou Province China. According to the results, the upload response time was about 0.8 s when the rate of upload request was 100 times/s, and the information query response time was about 1.2 ms when rate of query request was 1000 times/s. The performance of the system meets the requirements for its application in practical scenarios.

## Figures and Tables

**Table 1:** Analysis of the existing works in the literature.

Category	Representative Systems	Technology	Advantages	Disadvantages
Traditional system	Gao, G. et al. [27] Ga, A. et al. [28]	IoT	Enables food to be tracked and traced via IoT technology	Data security and reliability issues
	GS1 food safety traceability [29]	GS1standard; IoT, RFID	Enables the whole life cycle of the supply chain to be traceable; complies with GS1 standard	High cost of RFlD; data security and reliability issues
Blockchain-based system	Tian [30]	RFID, blockchain	Improves product traceability by covering the whole process of data gathering and information management	High cost of RFlD; on-chain storage capacity issues
	Huang, Y. et al. [31]	Blockchain; based on UTXO workflow	Prunes blockchain storage according to the expiration date of drugs	Quantitative assessment is not presented
	Chua, P. et al. [32]	Hyperledger fabric, EPCIS	Complies with EPCglobal Network standards;	Data explosion and privacy leakage problems
	Uddin, M. [33]	hyperledger fabric	Describes all of the aspects of the framework for drug traceability;	On-chain data explosion
Blockchain + off-chain system	Lin Q. et al. [34]	Ethereum, EPCIS	Collaborative management model of on-chain and off-chain data	Limited performance; depends on electronic cryptocurrency
	Yao, Q. et al. [35]	Ethereum, IPFS	Dual storage model to alleviate the blockchain’s storage pressure;	open participation, limited throughput, and high latency Depends on electronic cryptocurrency
	Zhang, L. et al. [36]	Fabric, IPFS	Dual storage model to alleviate the on-chain storage;	Lack of interactivity between nodes
	Wang, L. et al. [37] Zhang, X. et al. [38]	Fabric, IPFS	Dual storage model to alleviate the on-chain storage;	Quantitative assessment is not presented
Centralized database + cryptography primitive	LedgerDB [40], QLDB [41]	Cryptographic primitive and centralized database	Ensures data integrity and verifiability; high performance	Centralized ledger scheme: does not support consensus and smart contracts
Blockchain + database hybrid system	FalconDB [42]	Tendermint, MySQL, ADS	Data validation of client light nodes; transparent history query record	Limited API; dependent on incentive
	BigchainDB [43]	Tendermint, MongoDB	Ensures data integrity and tamper proof; high performance	Limited smart contract functionality

**Table 2:** EPCIS master data structure.

<EPCISMasterDataBody >
<VocabularyList>
<Vocabulary type = urn:cniotroot:vtype:master data type>
<VocabularyElementList>
<VocabularyElement id = master data ID>
< attribute id= ‘name’ >value</ attribute>
………
<attribute id = ‘name’>value</ attribute>
</VocabularyElement>
</VocabularyElementList>
</Vocabulary>
</VocabularyList>
</EPCISMasterDataBody>

**Table 3:** EPCIS Event Type.

Event Type	Meaning
ObjectEvent	It is related to the product and the operation fields, including ADD, OBSERVE, and DELETE
AggregationEvent	It describes the product aggregation, such as ‘package’
TransactionEvent	It describes the association or separation of a product with one or more businesses, such as ‘sale’ or ‘distributor’
TransformationEvent	It describes one or more product inputs, which are converted into a new product output (such as ‘process’)

**Table 4:** EPCIS event data structure.

<ObjectEvent> <eventTime>2020-03-17 14:00:00</eventTime> <eventTimeZoneOffset>+08:00</eventTimeZoneOffset> <epcList> <epc>694980901002201712300010007</epc> <epc>694980901002201712300010008</epc> </epcList> <action>OBSERVE</action> <productinfo:gtin>694980901002</productinfo:gtin> <productinfo:lot>20171230</productinfo:lot> <productinfo:productName>kiwi fruit</productinfo:productName> <productinfo:brandName>xifeng</productinfo:brandName> <productinfo:productionDate>2022-4-30</productinfo:productionDate> <productinfo:productionQuantity>2</productinfo:productionQuantity> <productinfo:packingSecification>bag</productinfo:packingSecification> <productinfo:itemExpirationDate>2022-01-06</productinfo:itemExpirationDate> <productinfo:uscID>86430111MA4L16J</productinfo:uscID> <productinfo:manufactureEnterprise>Guizhou Zhongkang Co., Ltd. </productinfo:manufactureEnterprise> </ObjectEvent>

**Table 5:** Off-chain EPCIS software environment.

Software	Version
Operating System	Window 10
Web server	Apache Tomcat 8.5.51
Database	MongoDB 3.6.15
Java Language	JDK 1.8

**Table 6:** Hyperledger fabric software environment.

Software	Version
Virtual Machine	Virtual Box 6.0.14
Operating System	Linux Ubuntu 16.04
Blockchain Platform	Hyperledger Fabric 1.4
Virtual Container	Docker 20.10.7
Go Language	Go 1.14.6

**Table 7:** Hardware environment.

Hardware	Version
CPU	Intel(R) Core(TM)i7-2600
Memory	8G DDR3 REG ECC
Hard Disk	SATA AHCI 200G
CPU Cache	8 MB

**Table 8:** Comparison between the proposed system and the other related works.

Representative Systems	On-Chain Data	IoT Device Authentication	Data Confidentiality	Data Interactivity	Performance
This work	Low	√	Permission management	√	100+ tps, upload time: 0.8 s~7 s query time ≈ 2 ms
[30]	High	×	Open participation and transparent	×	N/A
[32]	High	×	Permission management	√	N/A
[34]	Low	×	Open participation access permission control	√	Limited (upload time: 7~47s)
[35]	Low	×	Open participation and transparent	×	Limited (query time: 22 s for 243 MB)
[36]	Low	×	Permission management	×	High (25+ tps, upload time: ≈2.5 s)
[37]	Low	×	Permission management	×	N/A
[40]	/	×	Depend on Administrator	×	100k tps
[43]	High	×	Permission management	√	1000k tps

## Data Availability

Not applicable.
